# Supplementary material for: Label-aware distance mitigates temporal and spatial variability for clustering and visualization of single-cell gene expression data
Source: Commun Biol. 2024 Mar 14;7:326. doi: 10.1038/s42003-024-05988-y (PMC10940680; doi:10.1038/s42003-024-05988-y)
Supplement: Supplementary file 2 — Supplementary Information [file 42003_2024_5988_MOESM2_ESM.pdf]

Supplementary Materials

Supplementary Note 1: Visualization of distance correction

In the simulated data, the similarity between samples from the same or adjacent time points (e.g., “A2_d4_s1 and A2_d4_s2” and “S and S->A” in Figure R2c) is lost in the noisy version of the data (Figure R2a). The similarity is recaptured by LAD. Some small similarities are added to closer time points, which may be considered artifacts, but graph-based visualization methods (e.g., UMAP) can suppress this kind of noise.

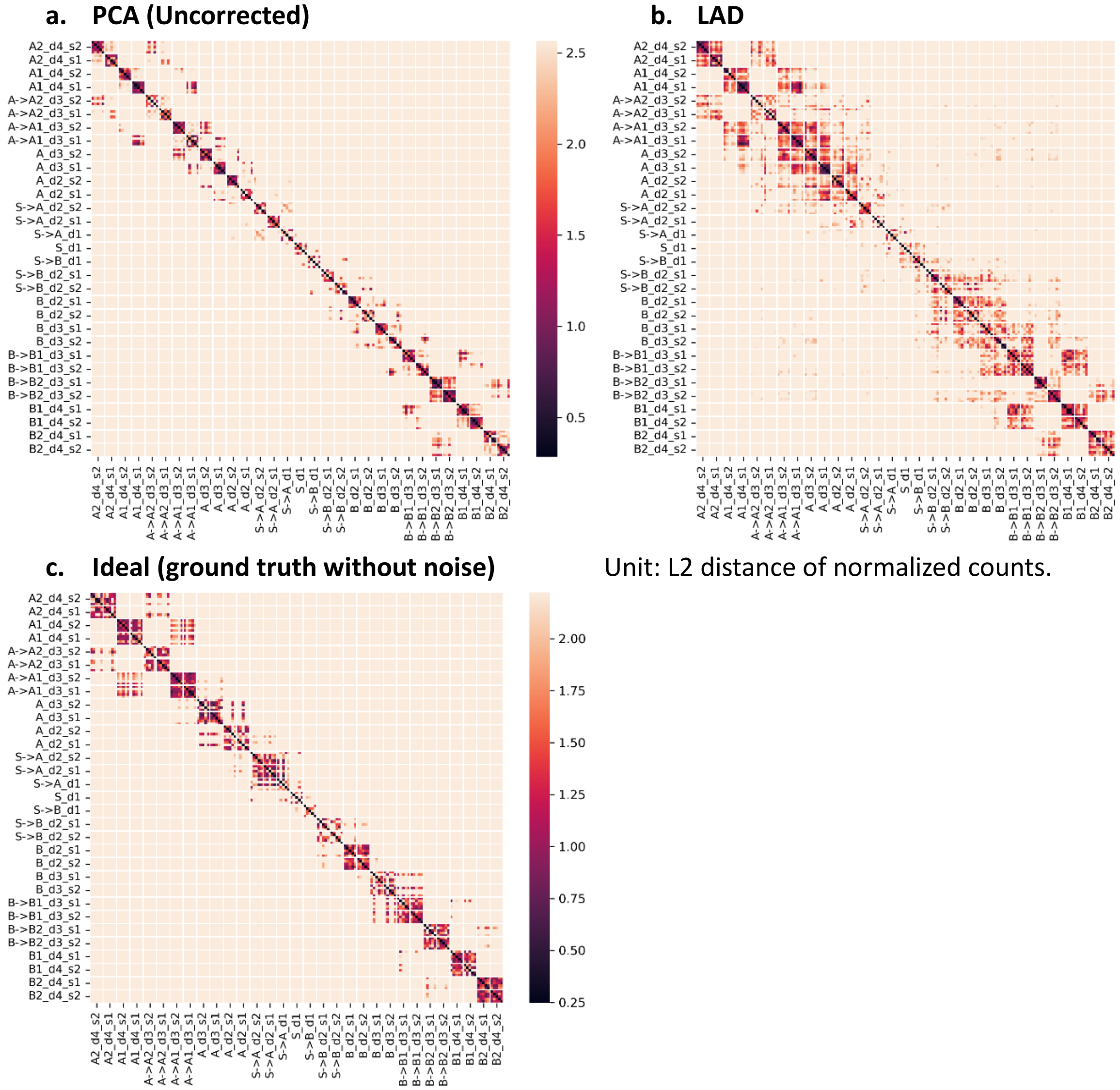

Supplementary Figure 1. L2 Distance of normalized counts (dimensionless) between simulated cells in each cell type of the uncorrected (a) and LAD corrected (b) data and ideal distance between cells before noise was added (c). Each cell type is downsampled to five cells per sample. The min and max cutoff value for the color bar is determined by 1/3 and 3 times the 0.9 percentile of all distances to emphasize the more similar cells. Shorter distances (darker color) indicate higher similarities. “d” stands for “day” and “s” stands for “sample” (for a specific day).

Supplementary Note 2: Trajectory/pseudotime inference

We used Monocle 3 to infer trajectories to show how the batch effect and different correction methods affect the results (**Supplementary Figure 2,3**). LAD shows more consistent distribution of pseudotime between repeats at the same time point, and an overall more monotonic increasing trend. The original publication identifies NFI transcription factors (genes: *Nfia*, *Nfib*, and *Nfic*) as candidate regulators of temporal patterning in the developing retina, characterized by a unimodal expression in RPCs over time. LAD faithfully illustrates this trend. By contrast, Seurat and Harmony produced pseudotime that are nearly indistinguishable between E14 and P5, and consequently created erroneous curves for NFI genes. The pseudotime with uncorrected data are acceptable notwithstanding some artifacts for E11, E12, and P14. However, the uncorrected data fail to capture the decreasing pattern of Nfia and Nfib, likely due to the artifact in P14. Limma shows oscillating expression of NFI genes, implying that the ordering of cells may be suboptimal. Overall, the label-aware correction strategy can lead to better trajectory/pseudotime inference results.

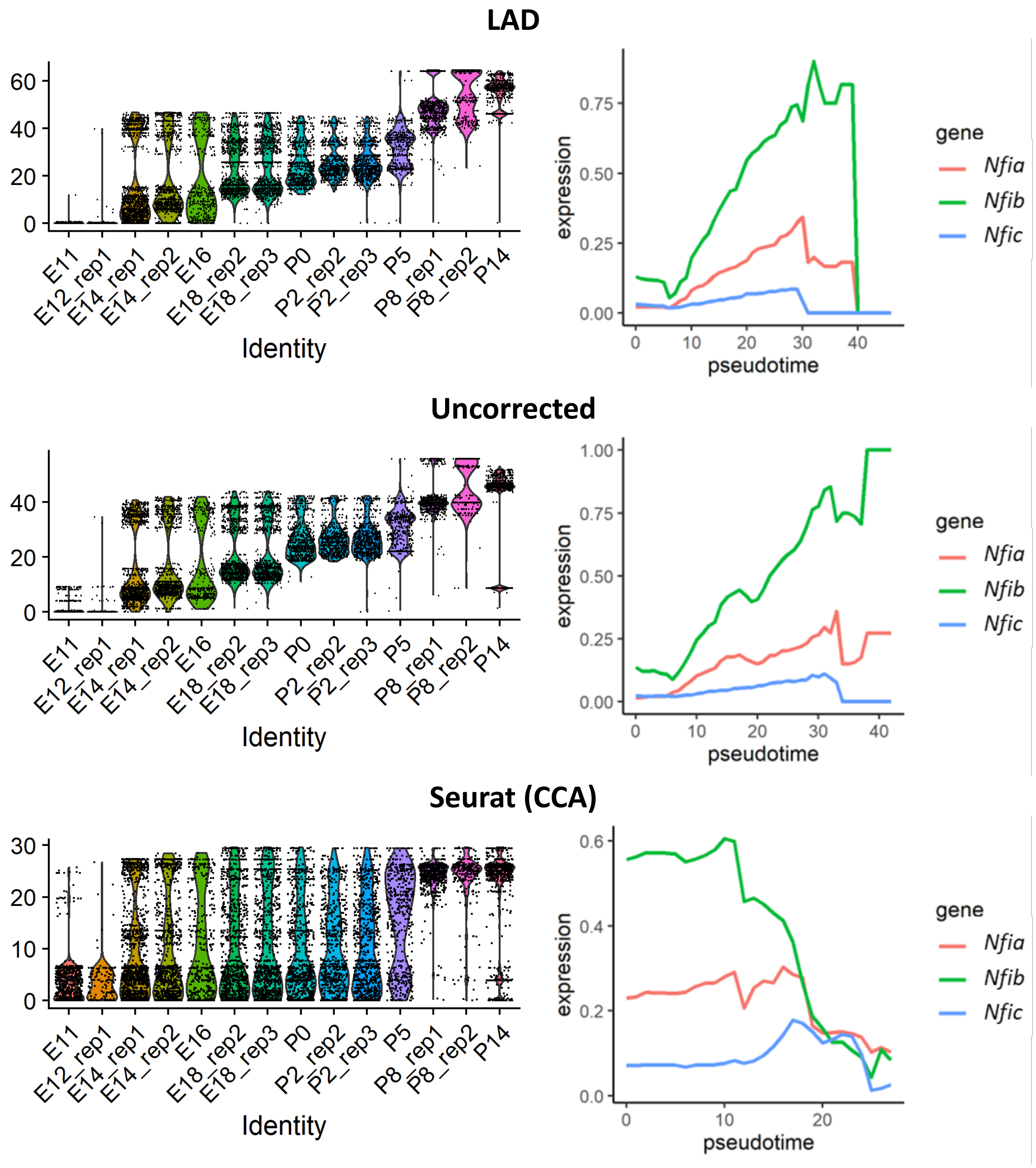

Supplementary Figure 2. Trajectory inference with Monocle 2 on uncorrected retina data and those corrected by LAD and Seurat (CCA). Left column: Distribution of inferred pseudotime within each sample. Right column: Average expression at each pseudotime in RPCs.

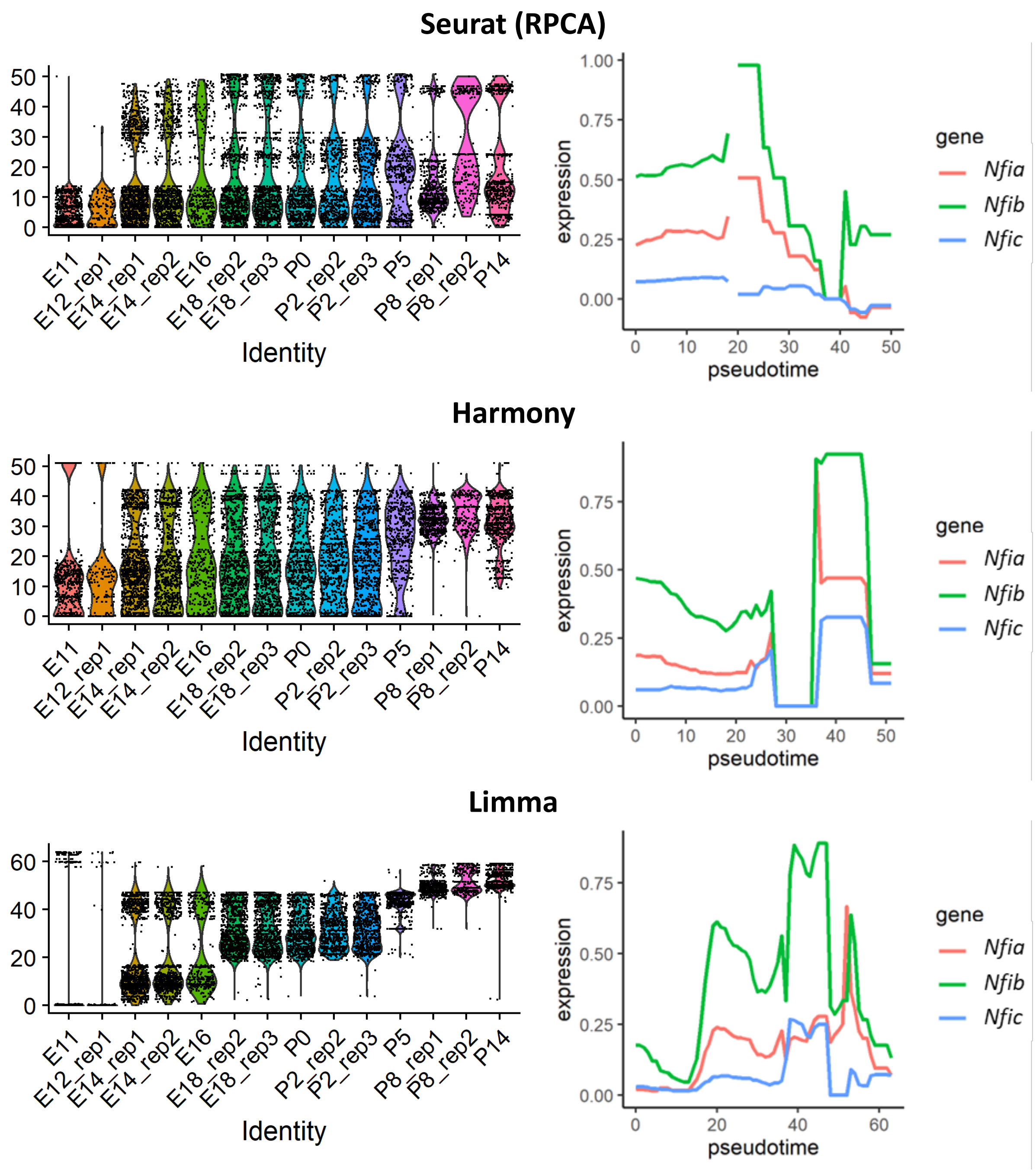

Supplementary Figure 3. Trajectory inference with Monocle 2 on retina data corrected Seurat (RPCA), Harmony, and Limma. Left column: Distribution of inferred pseudotime within each sample. Right column: Average expression at each pseudotime in RPCs.

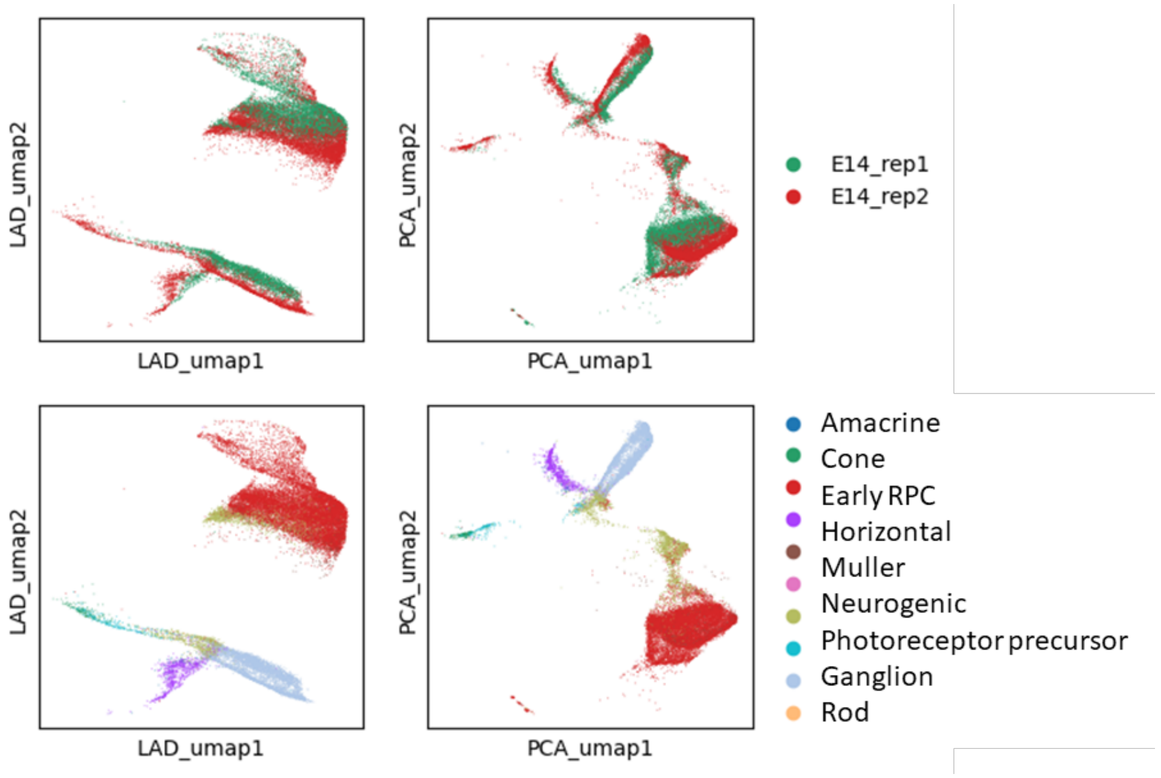

Supplementary Figure 4 Zoomed in view of remaining batch effect. ERPC cells from two replicates are integrated better than RGC, HC, and Photoreceptor progenitors.

Supplementary Note 3: Results on zebrafish embryo dataset

The original publication obtained a good mutual nearest neighbor graph without batch effect correction, suggesting that the batch effect is not large. Quantitative metrics shows that Limma has the top performance while PCA (uncorrected data) and LAD follows (**Supplementary Figure 4**). However, visually, all methods other than LAD and PCA mix pluripotent cells with differentiated cells, a sign of overcorrection. LAD fails to integrate 4-10 samples well, but cells from 10-24 samples are more reasonably clustered than other methods.

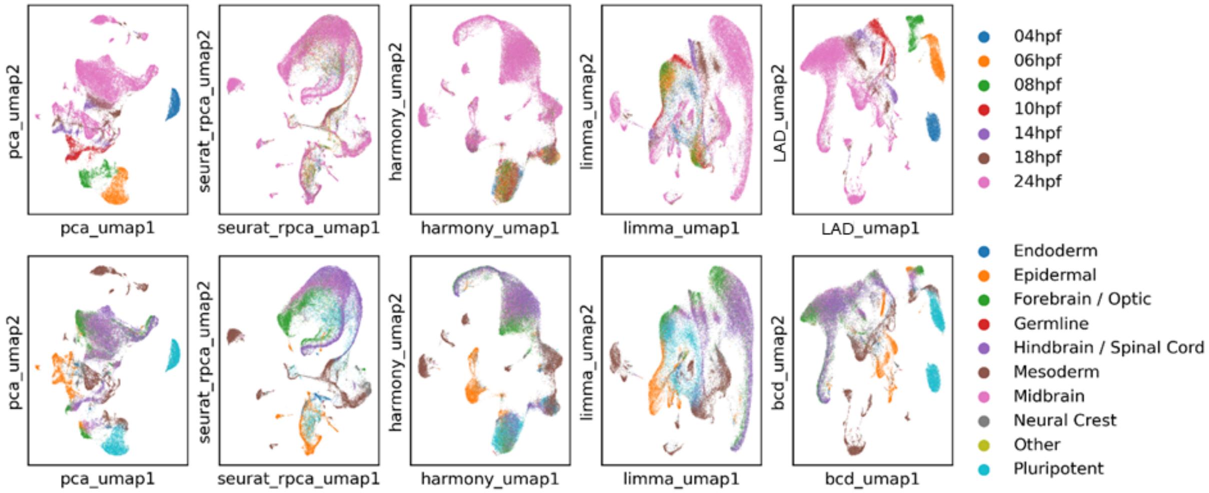

	Bio conservation		Batch correction		Aggregate score		
**Method**	Isolated labels	Silhouette label	Silhouette batch	Graph connectivity	Batch correction	Bio conservation	Total
**limma**	0.56	0.52	0.77	0.85	0.81	0.54	0.65
**LAD**	0.57	0.51	0.72	0.83	0.77	0.54	0.63
**pca**	0.57	0.51	0.71	0.83	0.77	0.54	0.63
**harmony**	0.50	0.48	0.79	0.70	0.74	0.49	0.59
**seurat_rpca**	0.50	0.49	0.78	0.52	0.65	0.49	0.56

Supplementary Figure 5. Results on Zebrafish embryo data. (Seurat Integration with CCA failed for running out of memory.)

Supplementary Note 4: Results on Human Fetal Lung Development Dataset

We compare the result of LAD and the other three methods in Supplementary Figure 2. As discussed in the main text, LAD delineates the gradual changes in macrophages along time and tissue location (**Supplementary Figure 5**). For Euclidean distance, the batch effect overcomes the similarity of cells. As a result, the mesenchymal cells from small airways and distal lung are separated into two clusters. Seurat integration removes the batch effect but creates an admixture of all time points and all locations, blurring the trajectory of development. In this particular case, Harmony also fails to remove such batch effect. Similar phenomena can also be observed in other cell types. Figures with detailed cell types do not fit on letter-sized paper. They are included in the supplementary files.

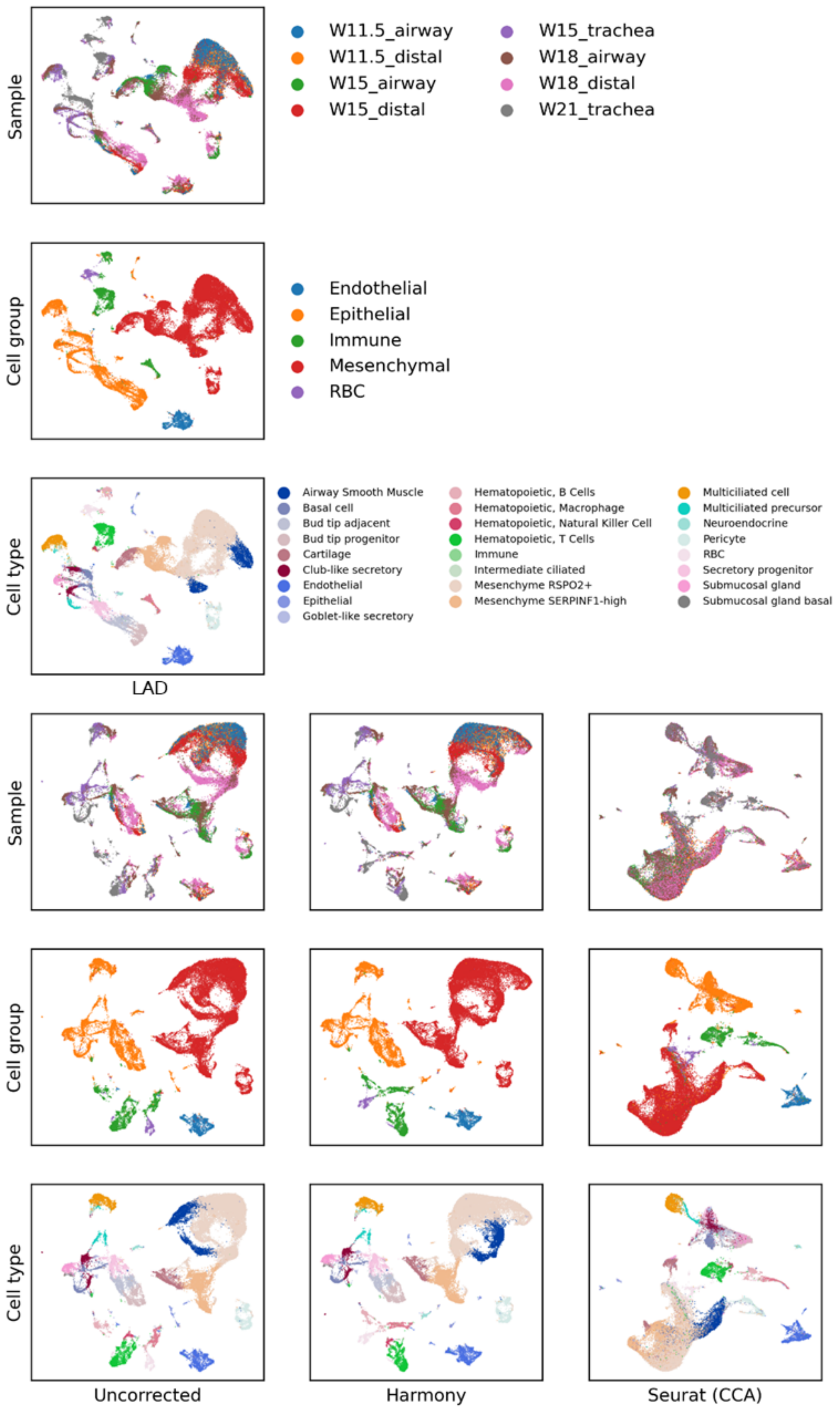

Supplementary Figure 6. Results on the human fetal lung development data colored by samples, major cell groups, and detailed cell types.

Supplementary Note 5: Sensitivity to hyperparameter l

We tested the sensitivity of LAD to hyperparameter l by benchmarking it with $l = 0.1$, 0.5, 1.0, 5.0, and 10.0 on the retina dataset. The resulting scores show no significant changes and consistently outperform other methods (**Figure 2** in the main text).

	Bio conservation		Batch correction		Aggregate score		
**Method**	Isolated labels	Silhouette label	Silhouette batch	Graph connectivity	Batch correction	Bio conservation	Total
LAD $l = 1$	0.64	0.58	0.85	0.95	0.90	0.61	0.73
LAD $l = 0.5$	0.63	0.59	0.85	0.95	0.90	0.61	0.73
LAD $l = 5$	0.64	0.58	0.85	0.95	0.90	0.61	0.72
LAD $l = 10$	0.64	0.58	0.85	0.95	0.90	0.61	0.72
LAD $l = 0.1$	0.63	0.58	0.84	0.95	0.90	0.61	0.72

Supplementary Figure 7. Sensitivity to hyperparameter l tested on the retina data.
